# A New Self-Made 16-Channel Capacitive Electrocardiography System: An Investigation and Validation for Non-Contact Electrodes

**DOI:** 10.3390/s25020445

**Published:** 2025-01-14

**Authors:** Jinru Yang, Tianjun Wang, Haipo Cui, Limin Sun

**Affiliations:** 1 School of Health Science and Engineering, University of Shanghai for Science and Technology, Shanghai 200093, China; yangjr725@163.com; 2 CAS Center for Excellence in Superconducting Electronics (CENSE), Shanghai Institute of Microsystem and Information Technology (SIMIT), Chinese Academy of Sciences, Shanghai 200050, China; wangtianjun@mail.sim.ac.cn

**Keywords:** non-contact electrode, cECG, motion artifacts, MNF, power line noise

## Abstract

Generally, the electrocardiography (ECG) system plays an important role in preventing and diagnosing heart diseases. To further improve the amenity and convenience of using an ECG system, we built a customized capacitive electrocardiography (cECG) system with one wet electrode, sixteen non-contact electrodes, two ADS1299 chips, and one STM32F303-based microcontroller unit (MCU). This new cECG system could acquire, save, and display the ECG data in real time. The bias feedback as a critical technique was routed to the left hand with the wet Ag/AgCl electrode, which could greatly suppress the power line noise. After all artifacts were removed, the ECG signals could be discerned clearly. We demonstrated that the ECG signals acquired with the capacitive electrodes were similar to those with the wet electrode. Thus, we successfully provide a new configuration for cECG monitoring at home or in a clinical setting.

## 1. Introduction

An electrocardiogram (ECG) is a noninvasive technology for measuring heart activity and is essential for diagnosing heart disorders. The ECG signals are recorded on the chest and generated from heartbeats. The normal ECG generally has a few electrodes, which is inadequate for the spatial information of heart activities. The disadvantages of using a small number of electrodes are poor spatial resolution and the inability to detect significant pathology.

To date, clinicians have paid great attention to the early warnings of heart disease. It is crucial to have a convenient device to monitor ECG signals over a long period. The best-matched electrode should be the non-contact electrode, although wet, semi-dry, and dry electrodes have better signal quality. However, the wet electrode needs conductive gel to maintain a moist surface and good contact with the skin. This preparation process is time-consuming, and the gel might dehydrate and stiffen after a while, leading to poor contact and degraded signal quality. The semi-dry electrode, which does not need conductive gel, relies on intrinsic conductive adhesives or saline to maintain effective skin contact. Likewise, maintaining stability over a rather long period remains a challenge. The dry contact electrode does not need to be moist on the surface but is pressed firmly against the skin to ensure adequate contact. Thus, all wet, semi-dry, and dry contact electrodes need to contact the skin, which can cause skin irritation or allergic reactions if long-term ECG monitoring is offered. The non-contact electrode does not need to touch the skin, which is perfectly matched for long-term ECG monitoring.

The non-contact electrode has developed at a fast pace in recent years. Based on different techniques, the non-contact electrode could be optical, capacitive, infrared, ultrasonic, microwavable, etc. Optical sensors and capacitive electrodes have been widely used in ECG monitoring. The optical sensor has been used to measure the color change in arterial blood in facial skin. With this technique, one can record the human heart rate by scanning the human face. However, this method is limited by the acquisition environment. Moreover, the measured results could only be used for assessing heart rate variability, making it difficult to show the most abnormal heart performance [1,2,3]. Doppler radar was expected to be widely used in measuring heart rate and respiration because of its small measurement constraints, versatility, and portability. However, the radar system is sensitive to electromagnetic fields, which means poor anti-interference ability [4,5]. Moreover, both optical and ultrasonic sensors only reflect the change in heart rate in ECG monitoring but lack other useful information, such as the phase in the millisecond scale.

The capacitive electrode is attractive due to its simplicity and practicality in ordinary life. This newly developed technique can capture the capacitance between the human body and the non-contact electrode. However, even for the ECG signals, this capacitance is weak and easily distorted by the body motion and environmental radio frequency interference [6], which is a big challenge. Thus, many new techniques have been used to improve the accuracy and stability of capturing tiny changes in capacitance. Many methods have been used to keep the ultra-high input impedance to reduce external interferences and avoid signal distortion, such as a bootstrap circuit [7,8], a parallel capacitor and resistor [9], and a reverse phase diode [10]. Other methods have been dedicated to improving the signal quality by using a feedback loop to reduce the common mode interference, a triaxial acceleration sensor to correct the motion artifacts, an output resistance divider to minimize the parasitic capacitance [9,11,12,13], or a shield against electromagnetic interferences and capacitive parasite coupling [8,14,15,16,17]. Therefore, a flexible non-contact electrode was designed to ensure a better fit for the subject and achieve improved capacitive coupling [18,19,20].

Although the capacitive ECG monitoring system has not been used in clinical applications, it has focused on ordinary living monitoring with non-contact electrodes bonding with a seat [21], a mattress [22], or some wearable devices [7,23]. However, such a system used only a few electrodes (generally 2–8), which was limited to acquiring spatial cardiac information.

Studies have shown that using more channels could improve the spatial resolution of cardiac electrical activity, which is critical for locating cardiac abnormalities, such as complex arrhythmias [24]. Moreover, the ECG monitoring system with more channels could obtain more comprehensive and accurate details during heart activity, which could help to identify potential heart problems such as myocardial hypertrophy and myocardial ischemia. In 2010, a capacitive electrocardiogram array, a 3 × 3 matrix, was placed on the back of a subject to measure phase-concerned electrical signals [25]. Recently, a standard 12-lead wearable ECG monitoring system was developed to support non-contact electrodes [26]. Later, an 8-channel ECG monitoring system with non-contact sensors located around the waist [27], a gain-adjustable 8-channel ECG acquisition system based on FPGA [28], and a 12-lead electrocardiogram device with ADS1293 were developed, respectively, to implement ECG monitoring [29].

In this study, we built a new 16-channel cECG system that could be used for daily ECG monitoring. The double shield design (the shield layer and the shield ring) was used for the electrode design against external environmental electromagnetic interferences to improve the signal-to-noise ratio (SNR). ADS1299 and STM32 were used to achieve the miniaturization of the multi-channel acquisition system, and a daisy-chain connection was used for all channels to provide a convenient approach for data acquisition on a large scale. We used 16 channels in our system, more than in previous studies, and used one single bias route to implement 16-channel unified bias feedback. This system was used to verify the non-contact electrodes in ECG monitoring by comparing them with the wet Ag/AgCl electrode.

## 2. Materials and Methods

**Figure 1:**
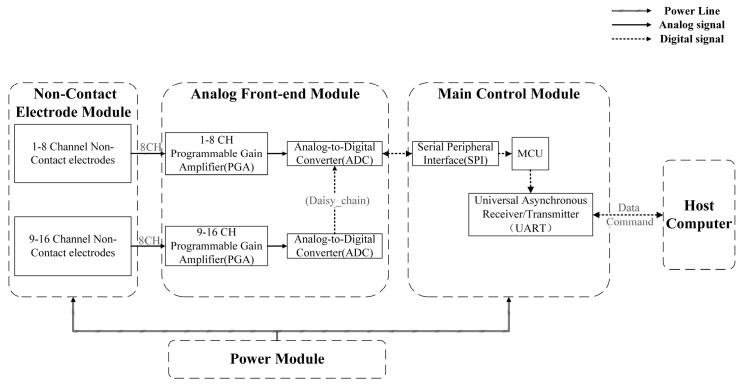
The architecture of the ECG acquisition system.

The architecture of the overall system is described in Figure 1. The customized ECG acquisition system consisted of a non-contact electrode module, an analog front-end (AFE) module, a main control module, a power supply module, and application software. The non-contact electrode module included 16 non-contact electrodes that acquired ECG signals by capacitive coupling. The AFE module used ADS1299 chips to sample the differential analog input and further interface with the main control module via the serial peripheral interface (SPI) protocol. The main control module was the bridge between the hardware and the software, which included setting up ADS1299 and sending the data package to the host. The application software was deployed in the host computer to display raw ECG data, filter the data in real time, and save the data. The power supply provided +5 V for the whole system, which used a chargeable lithium battery when measuring the ECG data.

### 2.1. The Non-Contact Electrode

**Figure 2:**
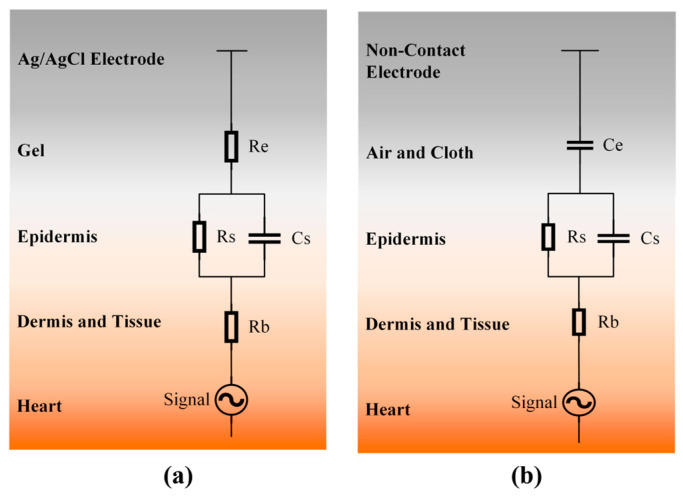
The equivalent circuit models of an Ag/AgCl electrode (**a**) and a non-contact electrode (**b**).

The non-contact electrode is similar to the traditional Ag/AgCl electrode. But they are also different. The Ag/AgCl electrode is sensitive to resistance in the body. The non-contact electrode is sensitive to the capacitance between the body and the non-contact electrode. Generally, the conductive gel acts as a small resistance and links the body and electrodes (Figure 2a) in the traditional electrode model. In the non-contact electrode model, there is a coupling capacitance $C_{e}$ between the epidermis and the metal plate of the electrode to conduct the signals (Figure 2b).

The coupling capacitance $C_{e}$ is defined by Equation (1).

(1) $$C_{e}=\frac{\varepsilon_{0}\varepsilon_{r}S}{d}=\frac{\varepsilon_{r}S}{4\pi kd}$$

where $\varepsilon_{r}$ is the relative dielectric constant depending on the materials between plates, $\varepsilon_{0}$ is the dielectric constant in the vacuum, *S* is the relative area between the two plates, *k* is the electrostatic force constant, which is defined as $\frac{1}{4\pi \varepsilon_{0}}$ in Coulomb’s law, and *d* is the distance between the plates. Thus, the size of the electrode plate, the ingredients of filled materials, and the thickness of the materials are the key factors impacting the signal quality.

**Figure 3:**
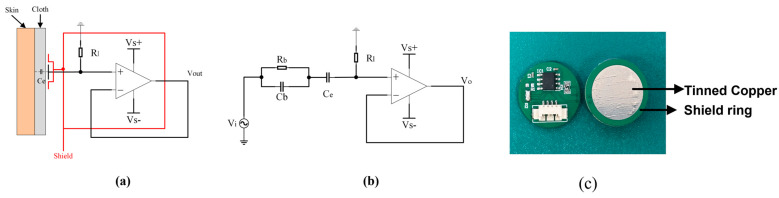
The design of the non-contact electrode: (**a**) the schematic circuit diagram, (**b**) the electrical model, and (**c**) the actual non-contact electrode.

The design of a capacitive non-contact electrode is shown in Figure 3a, which acquires ECG signals with ultra-high input impedance. This technique is critical for acquiring signals via capacitive coupling (Figure 3b) and suppressing the interference of body temporal varied impendence and the disturbance of environment background noise. One ultra-high resistor with 50 G Ohm was connected to the input of the operation amplifier, which acted as a voltage follower, resulting in a small output impendence.

Figure 3b shows the equivalent electrical model of the electrode. In this model, $R_{b}$ and $C_{b}$ are the equivalent resistance and capacitance and $Z_{b}$ is defined as the impedance of $R_{b}$ and $C_{b}$;${\;C}_{e}$ is the capacitance between the tinned copper plate of the non-contact electrode and the human epidermis; $R_{l}$ is the input load resistance of the designed model; and *s* is the complex frequency, which represents a stable state of the system. According to this electrical model, the transfer function of the non-contact electrode (Equation (2)) is as follows:

(2) $$G\left(s\right)=\frac{V_{O}}{V_{I}}=\frac{sC_{e}R_{l}}{1+sC_{e}R_{l}+sC_{e}Z_{b}}$$

(3) $$\underset{R_{l}\to \infty }{\lim}\frac{sC_{e}R_{l}}{1+sC_{e}R_{l}+sC_{e}Z_{b}}=\underset{R_{l}\to \infty }{\lim}\frac{sC_{e}}{sC_{e}}=1$$

(4) $$\underset{R_{l}\to 0}{\lim}\frac{sC_{e}R_{l}}{1+sC_{e}R_{l}+sC_{e}Z_{b}}=0$$

According to Equations (2)–(4), when $R_{l}$ approaches infinity, the input and output values of the system are fully correlated. When $R_{l}$ approaches zero, the input and output values of the system are fully uncorrelated. Given the above, the value of $R_{l}$ should be as large as possible. However, because $R_{l}$ acts as a form of bias resistance, a high bias resistance value makes it easy to introduce unwanted voltage errors for the precision operation amplifier, so $R_{l}$ should be limited by the bias current of the amplifier and supply voltage [16].

The current precision amplifier LMP7721(Texas Instruments, Dallas, TX, USA) has the industry’s lowest specified input bias current 3fA. According to the relevant description in the datasheet, due to the ultra-low input bias current of the LMP7721, the measurement error caused by the bias current is very small compared with other op-amp chips at ultra-high input impedance, and undesirable input voltage errors can be greatly avoided. Moreover, the characters of low-input reference voltage noise (8 nV/√Hz) and input-reference current noise (0.01 pA/√Hz) could maintain the high signal-to-noise ratio.

According to Equation (1), the capacitance is proportional to the plate area. Although the good quality of signals is related to the large plate area, the practical area is generally constrained due to the number of channels. We designed a circular electrode plate (Figure 3c) with a diameter of 18.8 mm. The four-layer PCB of the non-contact electrode included a signal layer, a ground layer, a shield layer, and an electrode plate layer. The electrode plate layer consisted of circular tinned copper, with a diameter of 15 mm as the one-side plate of the electrode, improving its corrosion resistance and electrical conductivity. To reduce the stray capacitance,$\;C_{stray}$, between the metal plate and the current amplifier, a shield ring with a width of 1.2 mm around the tinned copper electrode plate and the shield layer was applied to reduce environmental interference. Each electrode had its own shields, and the shields of all electrodes were not interconnected.

### 2.2. The Analog Front-End

The analog front-end circuit module used two ADS1299EVMs (Texas Instruments, Dallas, TX, USA) with the daisy chain connected to acquire data simultaneously. Each evaluation board contained 8 differential input channels with low-noise, 24-bit, high-precision synchronous analog-to-digital converters. The gain for each channel could be programmed from 1 to 24. The bias feedback technique was used to improve the common mode rejection: one channel was configured as the input of the bias feedback loop, and the bias signal was fed back to the human body. Specifically, all channels shared the same bias feedback loop. In our system, all ADS1299 chips used the same external clock to keep the synchronized sampling.

### 2.3. The Data Acquisition

**Figure 4:**
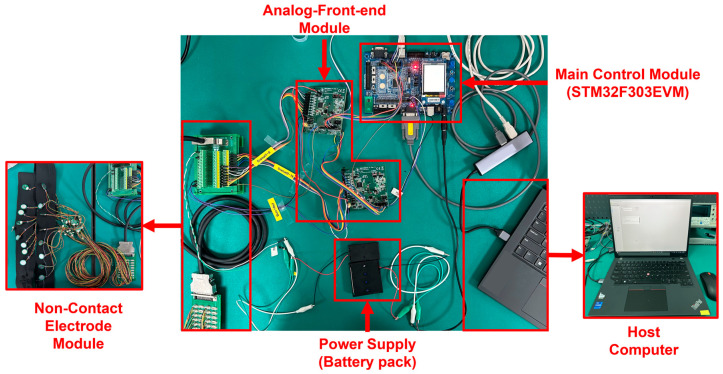
The customized cECG system.

As shown in Figure 4, the data acquisition module used the microcontroller (STM32F303VET6, STMicroelectronics, Geneva, CHE) to communicate with ADS1299 chips and the host computer. The general pipeline of this module was to receive data from ADS1299 chips via SPI and send the packaged data to the host computer via USART. The embedded program was written with C++ and burned into the MCU. The development environment was the STM32CubeIDE in Windows. The sampling rate was set manually through a user-friendly interface from the ECG system software. The actual control of the sampling rate depended on the configuration of the ADS1299 chip.

### 2.4. The ECG System Software

**Figure 5:**
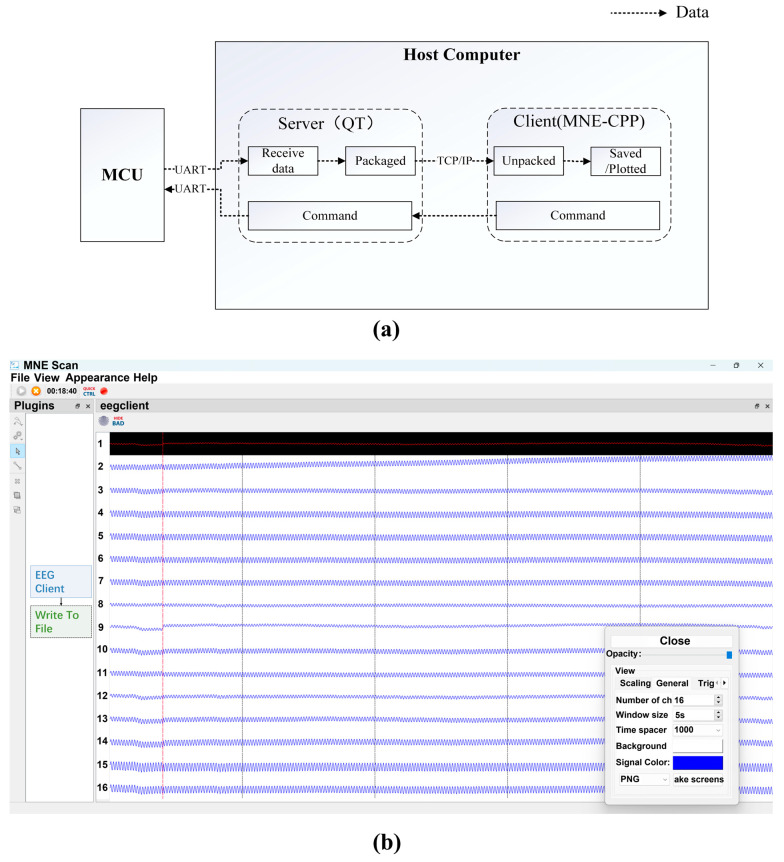
The acquisition software of the cECG system:(**a**) the architecture of data acquisition software, and (**b**) the GUI.

The ECG system software was developed with Qt C++. It included a server and a client. As shown in Figure 5, the server is an independent application that can acquire the ECG data from the hardware. The client was a newly made plugin of MNE-CPP, which is an open-sourced software initially published for babyMEG [30]. When up to date, it can be used for any device. The data were transferred from the server to the client via a TCP/IP socket. The ECG system software was deployed in a host computer (ThinkPad T16 Core i7 CPU @ 3.40 GHz, 32 GB RAM, 1 TB hard disk) in the Windows 11 operation system. However, this system software could also be deployed to any other OS due to Qt’s cross-platform property.

### 2.5. The Placement of Electrodes

**Figure 6:**
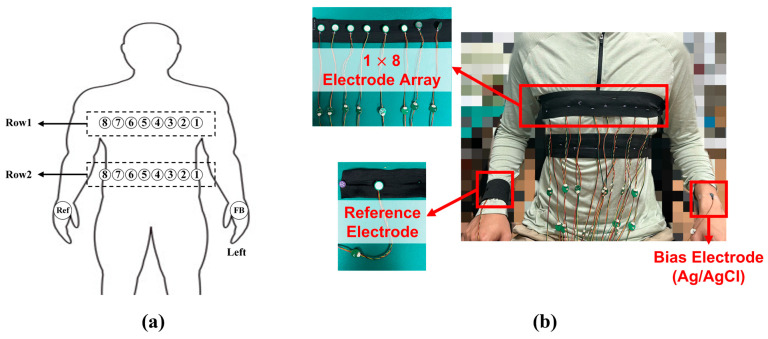
The distribution of multiple capacitive electrode locations: (**a**) the design and (**b**) the experiment.

In our system, the non-contact electrode array was placed on the chest (details in Figure 6). One reference electrode was placed on the back of the right hand. The bias feedback electrode was placed on the back of the left hand. Notably, the non-contact property makes the non-contact electrode unsuitable for the biased feedback drive. Therefore, the bias feedback electrode was a wet Ag/AgCl electrode. The other electrodes were all non-contact electrodes to acquire 16-channel ECG data.

## 3. Results

### 3.1. Measuring Small Signals

**Figure 7:**
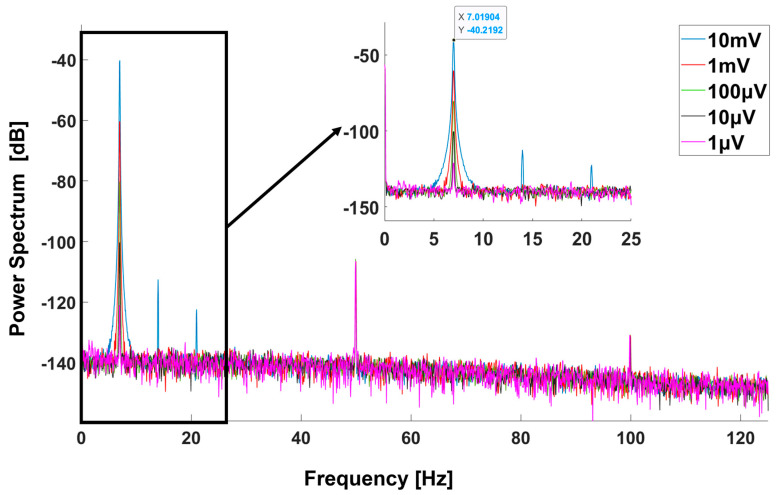
The spectra with different signals input.

We used a signal generator (Tektronix AFG 31000 SERIES, Arbitrary Function Generator, Tektronix, Beaverton, OR, USA) to simulate small signals with a controllable amplitude from 1 μV to 10 mV. An adjustable resistive voltage divider was serially connected to the signal generator. A simulated sine wave with 7 Hz was fed into our system. As shown in Figure 7, the cECG system can measure small signals as small as 1 μV. This sensitivity is sufficient for measuring ECG signals with amplitudes ranging from 0.1 mV to 2 mV.

### 3.2. Using the Bias Feedback Technique

**Figure 8:**
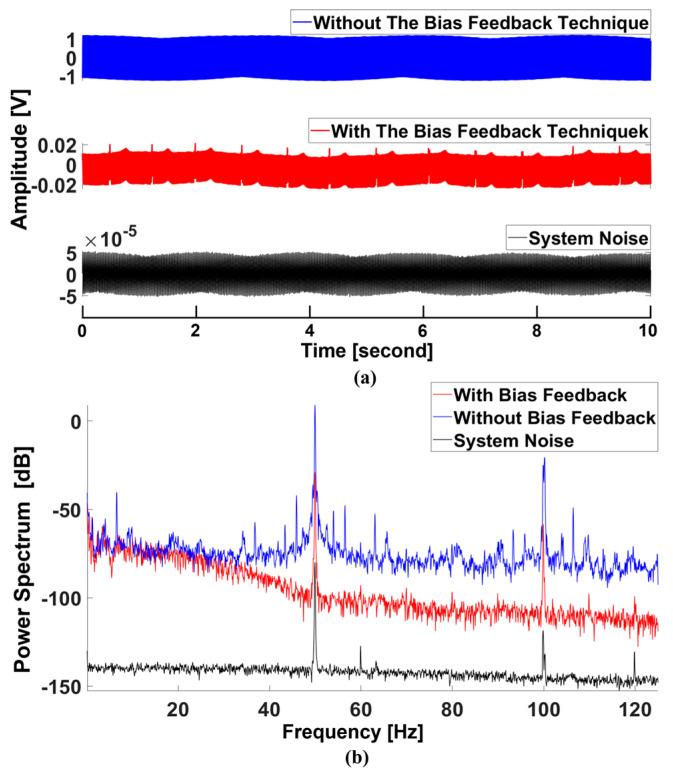
Comparison of cECG measurements with the bias feedback technique and without the bias feedback technique in the time domain (**a**) and in the frequency domain (**b**).

**Figure 9:**
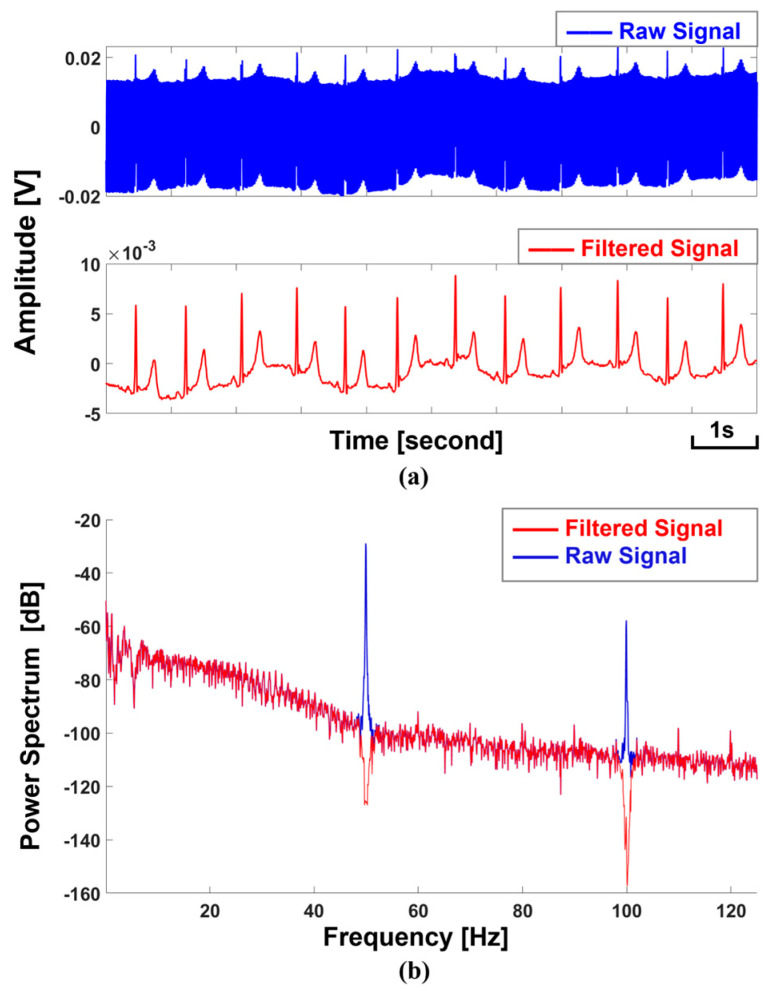
The power line artifacts were removed before and after applying two notch filters (50 Hz and 100 Hz). The comparisons are presented in the time domain (**a**) and the frequency domain (**b**).

During the acquisition of bioelectricity signals, the human body coupling with environmental noises resulted in significant common-mode interference, which generally was the reason for the saturated output signals. The bias feedback technique is considerably good at suppressing common-mode interferences and is crucial for removing environmental noise. To investigate the performance of this technique, we implemented three measurements: (a) measuring a subject with the bias feedback technique; (b) measuring a subject without the bias feedback technique; and (c) measuring the system noise without a subject. The results are shown in Figure 8. It is evident that the noise was very high when the bias feedback technique was not used. This high noise further caused the acquisition system to fail to capture the ECG signals. However, the power line noise was significantly suppressed if using the bias feedback technique. Thus, ECG signals could be discerned in the time domain (Figure 8a, middle). Besides the power line noise, other background noises (>40 Hz) were suppressed by approximately −80 DB. Despite these measures, the power line noise was still high. To address this, the notch filters of 50 Hz and 100 Hz were further applied to remove the residue power line noise (Figure 9a).

### 3.3. Removing Other Artifacts

**Figure 10:**
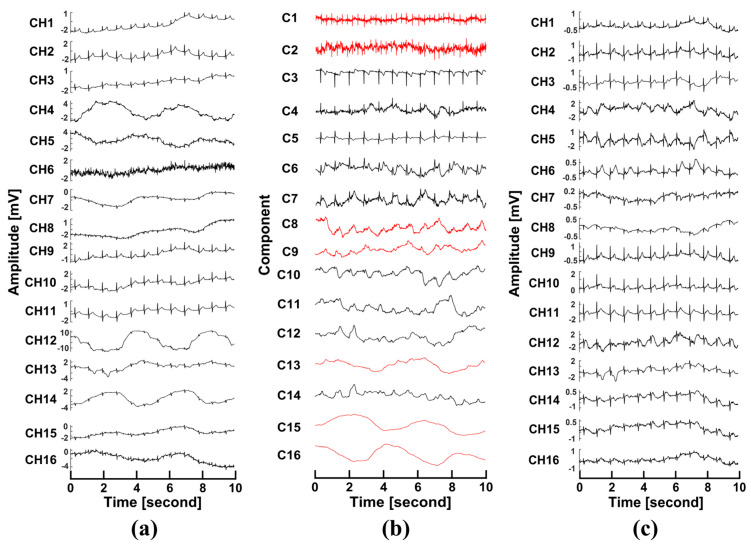
Artifact removal with MNF: (**a**) 16-channel data before the MNF algorithm; (**b**) independent components separated by the MNF algorithm (the red-marked components were identified as artifact components); and (**c**) the reconstructed 16-channel data.

Compared to wet electrodes, the non-contact electrodes were more sensitive to the subject’s body motion, heavy breathing, etc. The relative movements caused significant variations, which are called motion artifacts. These artifacts should be removed. The maximum noise fraction (MNF) method [31] is one of the blind source separation methods that could separate the noise and the signal in real time. The MNF method is based on the generalized singular value decomposition (SVD) that can separate the artifacts and the signals, assuming that the motion artifacts space is orthogonalized to the ECG signal space. We can obtain the artifact-removed ECG signals by selecting the artifact-related components, setting the value at zero, and reconstructing the left components. Figure 10a shows the 16-channel ECG data with the power-line interference removed. From applying the MNF method, the independent components (ICs) are listed in Figure 10b. There were high-frequency noise components (ICs 2, 3, 4, and 5) and motion artifact components (ICs 15 and 16), which should be removed. After reconstruction, ECG signals could be discerned clearly over all 16 channels, as shown in Figure 10c.

### 3.4. Estimating Materials for the Influence on Capacitance

The coupled capacitance depends on the material and the distance between the skin and the non-contact electrode. These factors are critical for acquiring high-quality signals. Therefore, we investigated several materials with different thicknesses using SNR (Equation (5)).

(5) $$SNR=10\log_{10}\frac{P_{signal}}{P_{noisy}}$$

We used cotton, polyester, and nylon as the experimental materials. There were nine setups, including 100% cotton with one layer, 100% polyester, 100% nylon, 70% cotton, 60% cotton, 60% polyester, 70% polyester, 100% cotton with two layers, and 100% cotton with three layers. The computed SNR was averaged over five times (Table 1).

The results show that 100% nylon, 100% cotton, and 100% polyester with one layer demonstrated better performance with a common mode rejection ratio (CMRR) above 70 dB. By comparing the experiments with 100% cotton, 70% cotton, and 60% cotton, we found that the experiment with 100% cotton provided the best performance. In the thickness test, the results (experiments 1, 8, and 9) proved that the CMRR decreased with the increased thickness. Table 1 shows that there were no significant differences in SNRs. The alpha level was at 0.05.

### 3.5. Comparing Wet and Non-Contact Electrodes

**Figure 11:**
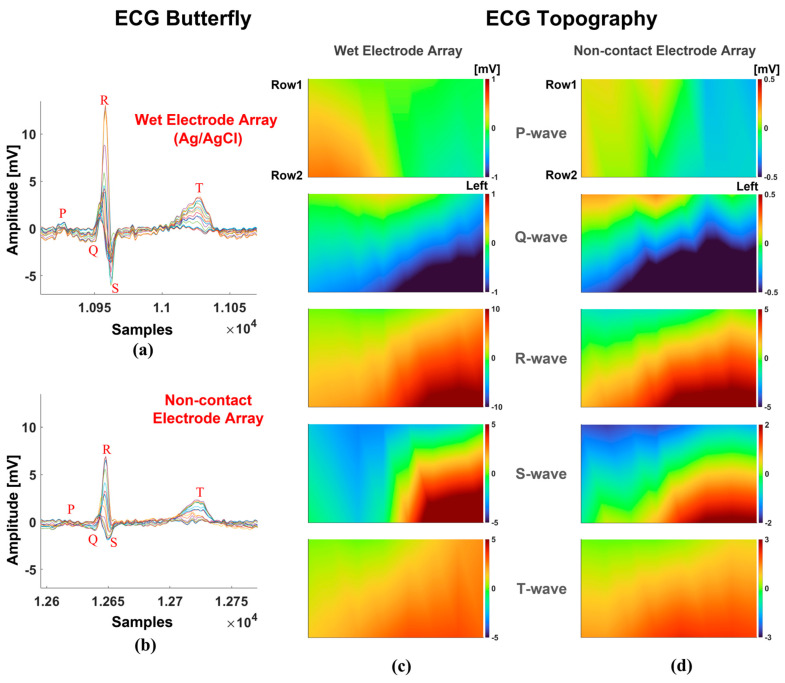
The comparison of wet and non-contact electrodes with the configuration of a 16-channel array: (**a**) the butterfly measured from the 16-channel wet electrode array; (**b**) the butterfly measured from the 16-channel non-contact electrode array; (**c**) the chest potentiostat at the P, Q, R, S, and T waves with the wet electrode array; and (**d**) the chest potentiostat at the P, Q, R, S, and T waves with the non-contact electrode array.

We used wet electrodes as the gold standard to validate the non-contact measurement [16,32,33]. We used a 16-channel array with wet/non-contact electrodes to monitor the depolarization and repolarization of the human heart individually. With this setup, we could observe the current flow temporally and spatially, which provides more information on heart diseases. Figure 11 presents the QRS waves acquired via wet/non-contact electrodes. The results show a similar process for the depolarization and repolarization of heartbeats.

## 4. Discussion

Recently, researchers have paid more attention to the non-contact applications of ECG. This is very convenient for clinical applications and more suitable for home monitoring. However, the electrode’s size and noises are challenges.

There is a trade-off between the signal quality and the electrode size. As a capacitive electrode, the size of the plate is a critical factor for the quality of ECG signals. The bigger the plate, the better the signal quality we obtained. However, if we designed a system with large-scale electrodes, the size of the electrode plate would be restricted. In our study, we used a circular tinned copper with a diameter of 15 mm, which proved feasible for an ECG system. The recorded signals were stable and of good quality, verified by the contrast measurements (wet vs. non-contact electrodes).

It was not surprising that we had a significant power line noise since the non-contact electrodes were easily coupled with environmental interferences. We confirmed that the bias feedback technique was efficient in suppressing the power line noise. Besides this, using 50 Hz and 100 Hz notch filters could further reduce the power line noise. We also investigated the system’s noise with the small signal input and demonstrated that our system achieved a low noise level capable of acquiring ECG signals.

Because the signals were acquired through capacitive coupling, the system was more sensitive to body movement. We noticed that strong movements (i.e., twisting, jumping, etc.) and frequently changing motion states should be restricted during the measurement. In such a way, we can avoid signal saturation and distortion. Large motion artifacts are a significant disadvantage of using non-contact electrodes in many applications. Therefore, removing motion artifacts is critical. We assumed that the motion artifact space was orthogonalized to the signal space. Thus, blind source separation (BSS) methods can be applied. However, the recorded artifacts were more complicated due to other artifacts such as chest fluctuation during breathing, slight limb movement, the movements of nearby subjects, etc. The separated artifacts were generally distributed in more than one component. We used the MNF method and selected more than one IC (marked as noises) to remove motion artifacts. After reconstruction, the heartbeats were discerned. We believe that other similar BSS methods could achieve the same results. Due to the sensitivity to motion, the respiratory information can be selectively extracted from the raw data, which implies that the heartbeat and the respiratory data could be analyzed simultaneously in future applications.

We investigated the performance of materials between the skin and the non-contact electrode. We found no significant difference when subjects wore different material clothes, which means ordinary clothes can be worn during the measurement. However, given the slightly improved SNR values, we suggest that the subject wears thin clothes made of 100% cotton.

We also compared wet and non-contact electrodes. The non-contact electrode array and the wet electrode array had discrepancies in the measured signal amplitude due to the different physical principles of the electrodes. However, the measurement showed that the non-contact electrodes demonstrated excellent performance of depolarization and repolarization in one heartbeat cycle, similar to the measurements with wet electrodes. Thus, the non-contact electrode is reliable and could replace the wet electrode with routine ECG monitoring.

## 5. Conclusions

We investigated the non-contact electrodes with a customized ECG system. This system consisted of 16 non-contact electrodes, one wet Ag/AgCl electrode, the analog front-end, the MCU, and the acquisition software, which could record, save, and display ECG signals. We proved that the bias feedback technique was critical to suppressing the power line noise and confirmed that ECG signals were discerned clearly after the power line noise was suppressed and the motion artifacts were removed. By comparing different materials placed between the skin and the non-contact electrode, we concluded that there was no specific requirement for the subject’s clothes. By comparison, we found that non-contact electrodes were able to acquire the correct ECG signals. In summary, we provide a simple ECG system to validate non-contact electrodes and acquire ECG signals successfully.

## Figures and Tables

**Table 1:** Estimates of the materials and the thickness.

Number	Ingredient	Layer	SNR
1	100% Cotton	1	77.37 dB
2	100% Polyester	1	72.32 dB
3	100% Nylon	1	74.83 dB
4	70% Cotton	1	71.82 dB
5	60% Cotton	1	68.93 dB
6	60% Polyester	1	71.02 dB
7	70% Polyester	1	72.13 dB
8	100% Cotton	2	71.04 dB
9	100% Cotton	3	67.97 dB

## Data Availability

The data that support the findings of this study are available from the corresponding authors upon reasonable request.
